# Information Hiding Based on Statistical Features of Self-Organizing Patterns

**DOI:** 10.3390/e24050684

**Published:** 2022-05-12

**Authors:** Loreta Saunoriene, Kamilija Jablonskaite, Jurate Ragulskiene, Minvydas Ragulskis

**Affiliations:** 1 Center for Nonlinear Systems, Kaunas University of Technology, Studentu 50-146, LT-51368 Kaunas, Lithuania; jurate.ragulskiene@ktu.lt (J.R.); minvydas.ragulskis@ktu.lt (M.R.); 2 Faculty of Mathematics and Natural Sciences, Kaunas University of Technology, Studentu 50, LT-51368 Kaunas, Lithuania; kamilija.jablonskaite@ktu.edu

**Keywords:** predator-prey model, self-organizing pattern, Shannon entropy, image hiding

## Abstract

A computational technique for the determination of optimal hiding conditions of a digital image in a self-organizing pattern is presented in this paper. Three statistical features of the developing pattern (the Wada index based on the weighted and truncated Shannon entropy, the mean of the brightness of the pattern, and the *p*-value of the Kolmogorov-Smirnov criterion for the normality testing of the distribution function) are used for that purpose. The transition from the small-scale chaos of the initial conditions to the large-scale chaos of the developed pattern is observed during the evolution of the self-organizing system. Computational experiments are performed with the stripe-type patterns, spot-type patterns, and unstable patterns. It appears that optimal image hiding conditions are secured when the Wada index stabilizes after the initial decline, the mean of the brightness of the pattern remains stable before dropping down significantly below the average, and the *p*-value indicates that the distribution becomes Gaussian.

## 1. Introduction

Steganography is the art of hiding a secret information in non-secret media such as a digital image, an audio or video recording, a printed document, etc. [1]. The purpose of steganography is not only to conceal the secret message, but also to hide the fact of secret communication itself. Different types of digital images (including natural and artificial patterns and textures) has been exploited for the secret communication. Although numerous steganographic techniques employ conventional grayscale or colour photographs for the encoding of the secret information [1,2,3], different options for a carrier image can be found in the scientific literature. For example, the investigation proposed in [4] suggests to embed the secret data in complex blocks of digital photographs using block patterns. The steganographic technique concealing the secret information in printed bicolor documents is presented in [5]. A novel image steganography scheme based on a color assimilation illusion is proposed in [6], where a synthesized image containing a grayscale background and a saturated color line grid can be perceived as a color image. The authors of [7] suggest to choose the most suitable candidates for the cover image from the whole set of images based on a relative entropy and a histogram.

Pattern formation has been attracting the attention of researchers in different branches of science since the middle of the twentieth century [8]. Self-organisation can be observed on all scales of biological life (biological networks, animal patterns, spatial vegetation patterns, microbial communities, cells communication) [9,10,11,12,13], in chemistry (reaction–diffusion systems, Turing patterns, electrochemical synthesis) [14,15,16], in physics (plasmas, crystals, magnetic granular media, semiconductor resonators, solitons) [17,18,19,20], in computers and engineering (cellular automata, control of multirobot swarms and self-assembling microrobots, etc.) [21,22,23].

Moreover, self-organizing patterns are used for hiding and communicating secret visual images. For example, a digital fingerprint image is employed as the initial input for the evolution of a pattern using a model of reaction–diffusion cellular automata [24]. Schemes for the concealing of the secret visual information in self-organizing patterns can be implemented employing different models of dynamical systems. For example, a communication scheme based on evolutionary spatial 2 × 2 games is introduced in [25], where self-organizing patterns are induced by complex interactions between competing individuals. Similar digital image communication schemes employ self-organizing patterns emerging from arrays of competitively coupled nonlinear maps [26] or from the breakup of spiral waves [27]. Models described by the systems of partial differential equations can also be used for the secret communication task. A typical example is a steganographic algorithm based on the patterns evolving from the Beddington-deAngelis-type (BDA) predator-prey model with self- and cross-diffusion [28], introduced in [29]. This algorithm is designed for hiding dichotomous secret images in the self-organizing pattern developed from the perturbed initial conditions [29].

The initial conditions of the coupled fields of preys and predators in the BDA predator-prey model are perturbed around their stationary states in order to induce the unstable breaking waves of Turing instability. In other words, the small-scale spatial chaos in the initial conditions (predetermined by the random number generator) is evolving into the large-scale spatial chaos (predetermined by the type of the self-organizing pattern). However, this transition is far from being trivial. Nonlinear processes of self- and cross-diffusion in the BDA predator-prey model do result into long and complex transient processes until the fully developed patterns can be clearly observed in the distribution of preys and predators [28].

On the other hand, it appears that the difference image between the self-organizing pattern originated from the perturbed and non-perturbed initial conditions becomes non-interpretable after long transients [29]. Therefore, an efficient and coherent application of these image hiding algorithms based on self-organizing patterns does require a clear and comprehensible strategy for choosing the stopping criterion in the evolution of those patterns.

The main objective of this paper is to propose a statistical indicator which could be related to the optimal information hiding features in self-organizing patterns. The paper is organized as follows. The BDA predator-prey model with self- and cross-diffusion, the secure communication system based on self-organizing patterns and statistical indicators for the evaluation of the image complexity are presented in Section 2. Optimal information hiding in different types of self-organizing patterns is discussed in Section 3. Concluding remarks are given in the final section.

## 2. Preliminaries

### 2.1. Beddington-DeAngelis-Type Predator-Prey Model with Self- and Cross-Diffusion

Governing equations of the BDA predator-prey model with self- and cross-diffusion read [28]:

(1) $$\begin{array}{ccc} \frac{\partial N}{\partial t} & = & r\left(1-\frac{N}{K}\right)N-\frac{\beta N}{B+N+wP}P+D_{11}\nabla^{2}N+D_{12}\nabla^{2}P,\\ \frac{\partial P}{\partial t} & = & \frac{\varepsilon \beta N}{B+N+wP}P-\eta P+D_{21}\nabla^{2}N+D_{22}\nabla^{2}P, \end{array}$$

where *t* denotes time; *N* and *P* denotes the densities of preys and predators respectively; β stands for a maximum consumption rate; *B* is a saturation constant; *w* is a predator interference parameter; η is a per capita predator death rate; ε represents the conversion efficiency of food into offspring. $D_{11}$ and $D_{22}$ denote self-diffusion coefficients. The cross-diffusion coefficient $D_{12}$ represents the tendency of a prey to keep away from a predator while $D_{21}$ represents the tendency of a predator to chase its prey. The operator $\nabla^{2}=\frac{\partial^{2}}{\partial x^{2}}+\frac{\partial^{2}}{\partial y^{2}}$ is the Laplacian operator in the two-dimensional space. $D_{11}\nabla^{2}N$ and $D_{22}\nabla^{2}P$ are self-diffusion terms, which imply the movements of individuals from a higher to lower concentration region; $D_{12}\nabla^{2}P$ and $D_{21}\nabla^{2}N$ are cross-diffusion terms that biologically imply the counter-transport [28].

Model in Equation (1) is analysed under non-zero initial conditions and Neumann boundary conditions. Densities of preys and predators

(2) $$N\left(x,y,0\right)>0;P\left(x,y,0\right)>0$$

are set in a rectangular domain $\left(x,y\right)\in \Omega =\left[0,L_{x}\right]\times \left[0,L_{y}\right]$, where $L_{x}$ and $L_{y}$ is the size of the system in the directions of x- and y-axis.

Neumann, or zero-flux, conditions are set on the boundary:

(3) $$\frac{\partial N}{\partial n}=\frac{\partial P}{\partial n}=0;\left(x,y\right)\in \partial \Omega ,$$

where *n* is the outward unit normal vector of the smooth boundary ∂Ω.

In the absence of diffusion, the model has a single non-trivial stationary state $\left(N^{*},P^{*}\right)$ (coexistence of preys and predators), where [28]:

(4) $$\begin{array}{ccc} N^{*} & = & \frac{1}{2rw\varepsilon }K\left(rw\varepsilon -\varepsilon \beta +\eta \right)+\frac{1}{2rw\varepsilon }\sqrt{K^{2}{\left(rw\varepsilon -\varepsilon \beta +\eta \right)}^{2}+4rKw\varepsilon \eta B};\\ P^{*} & = & \frac{\left(\beta \varepsilon -\eta \right)}{w\eta }N^{*}-\frac{B}{w}. \end{array}$$

### 2.2. The Numerical Model and Types of Self-Organizing Patterns

Standard five-point approximation for 2D Laplacian with the zero-flux boundary conditions is used to find the solution of Equation (1). The densities of preys and predators $\left(N_{ij}^{n+1},P_{ij}^{n+1}\right)$ at the time moment $\left(n+1\right)\tau$ at grid position $\left(x_{i},y_{j}\right)$ read [28]:

(5) $$\begin{array}{ccc} N_{ij}^{n+1} & = & N_{ij}^{n}+\tau D_{11}\Delta_{h}N_{ij}^{n}+\tau D_{12}\Delta_{h}N_{ij}^{n}+\tau f\left(N_{ij}^{n},P_{ij}^{n}\right),\\ P_{ij}^{n+1} & = & P_{ij}^{n}+\tau D_{21}\Delta_{h}N_{ij}^{n}+\tau D_{22}\Delta_{h}P_{ij}^{n}+\tau g\left(N_{ij}^{n},P_{ij}^{n}\right), \end{array}$$

where the Laplacian is

(6) $$\begin{array}{c} \Delta_{h}N_{ij}^{n}=\frac{N_{i+1,j}^{n}+N_{i-1,j}^{n}+N_{i,j+1}^{n}+N_{i,j-1}^{n}-4N_{i,j}^{n}}{h^{2}}. \end{array}$$

At time moment t=0, the system is placed into the stationary state $\left(N^{*},P^{*}\right)$ perturbed by a random perturbation $\left[\tilde{N}\right]$ (induced by a random number generator or a logistic map) [29]. A long transient process is required for the system to evolve into a steady or time-dependent state. Different sets of the model parameters lead to the distinct types of the evolved patterns: stripe-type patterns, spotted patterns, the mixture of spotted and stripe-type patterns or the unstable spiral wave patterns [28]. Note that different initial perturbations of the stationary states of preys and/or predators do result into the patterns of the same type (stripes, spots, stripes–spots or spiral wave) but with a different distribution of stripes, spots or waves [28].

### 2.3. A Secure Communication System Based on Self-Organizing Patterns

**Figure 1:**
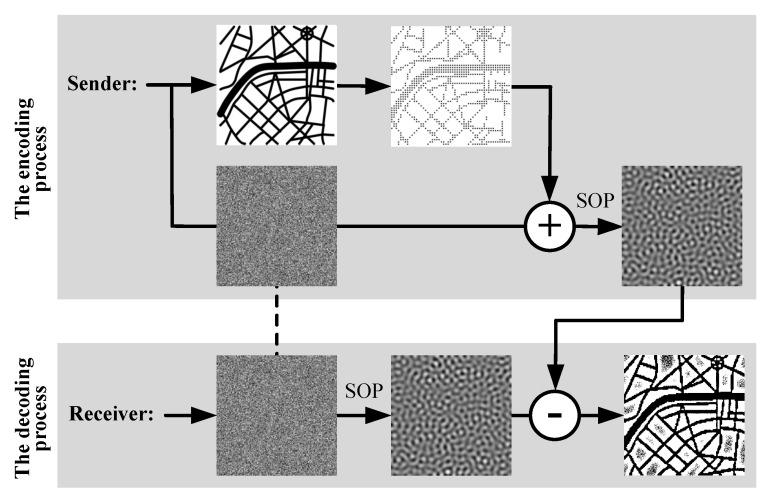
A schematic representation of the secure communication system based on the formation of self-organizing patterns. The encoding process: the Sender constructs the dot skeleton representation of the secret image $\left[M\right]$ and generates the random distribution of preys $\left[N\right]{|}_{t=0}$. The plus sign corresponds to the perturbation of the initial random distribution; the arrow marked by SOP represents the evolution of the self-organizing pattern. The decoding process: the dashed line indicates that the Receiver must generate an exact copy of the random distribution of preys $\left[\tilde{N}\right]$. The arrow marked with SOP represents the evolution of the self-organizing pattern. The secret image is decoded by computing the difference between the evolved and the received patterns (what is represented by the minus sign in the diagram).

A secure communication system based on the formation of self-organizing patterns introduced in [29] can be described by the schematic diagram depicted in Figure 1.

The schematic representation of the encoding process is presented at the upper part of Figure 1. Initially, the model parameters (the number of time steps *n*, values of parameters ε≪1 and δ≪ε≪1, the set of random numbers $\left[\tilde{N}\right]$ distributed on the interval $\left[-\frac{\varepsilon }{2};\frac{\varepsilon }{2}\right]$) should be selected and fixed between the Sender and the Receiver. Then, the Sender generates the dot-skeleton representation of the secret image and constructs the mask matrix $\left[M\right]$ [29]. The next step is the perturbation of the initial density of preys $\left[N\right]{|}_{t=0}$ according to the following rule [29]:

(7) $$\left[N\right]{|}_{t=0}=N^{*}\cdot \left[1\right]+\left[\tilde{N}\right]+\delta \cdot \left[M\right],$$

where δ is a small constant that guarantees that the mask perturbation is smaller than the noise used for the perturbation of initial conditions (δ≪ε); $\left[M\right]$ is the mask matrix comprising ones at those positions where the initial random density of preys $\left[\tilde{N}\right]$ is increased by δ — and zeros where the random density of preys $\left[\tilde{N}\right]$ is kept unchanged. The numerical scheme presented in Equations (5) and (6) is used to evolve the perturbed initial density of preys $\left[N\right]{|}_{t=0}$ for *n* time steps. Finally, the digital image of the evolved pattern is sent to the Receiver. As mentioned previously, the initial perturbation $\left[\tilde{N}\right]$ (Equation (7)) does not change the type of the pattern. However, the initial perturbation $\left[\tilde{N}\right]$ does change the local distribution of stripes, spots, or spiral waves [28]. The mask representation $\delta \cdot \left[M\right]$ (Equation (7)) does not change the local distribution of stripes, spots, or spiral waves unless δ is smaller than the noise used for the perturbation of initial conditions (δ≪ε) [29]. Local deformations of the elements of the pattern help to hide the secret image. However, a different distribution of stripes, spots, or spiral waves would compromise the image hiding algorithm [29]. Therefore, it is important to ensure that the initial perturbation $\left[\tilde{N}\right]$ must be kept identical for both patterns generated by the Sender and the Receiver [29].

The decoding process is depicted at the lower part of the schematic diagram in Figure 1. Initially, the Receiver sets the values of the model parameters (the number of time steps *n*, parameters ε≪1 and δ≪ε≪1, generates the set of random numbers $\left[\tilde{N}\right]$ distributed on the interval $\left[-\frac{\varepsilon }{2};\frac{\varepsilon }{2}\right]$). Then, the initial densities of preys are perturbed

(8) $$\left[N\right]{|}_{t=0}=N^{*}\cdot \left[1\right]+\left[\tilde{N}\right]$$

and the numerical scheme in Equations (5) and (6) is used to evolve the pattern for *n* time steps. Note that the random noise perturbations $\left[\tilde{N}\right]$ in Equations (7) and (8) are the same. The secret image is revealed in the form of the difference image between the evolved and the received patterns. Image enhancement techniques can be applied to the difference image in order to obtain the dichotomous representation of the secret image.

The information hiding capacity is predetermined by the dot-skeleton representation of the secret image [29]. A thorough computational analysis has shown that a clearance of 7 pixels between two adjacent pixels ensures the development of a well-interpretable line in the difference image [29]. Two separate lines can be clearly interpreted in the difference image when the distance between those lines is 20 pixels [29]. Thus, the maximal information hiding capacity of the scheme based on the BDA model is predetermined by the maximal distance between pixels constituting a line, and the minimal distance between two separable lines (7 and 20 pixels). Of course, the hiding capacity also depends on the shape of the dichotomous secret image represented by a pattern of lines. The distances between pixels in the dot-skeleton representation of the secret image just determine the maximal resolution of the secret image [29].

As mentioned previously, the main objective of this paper is to design statistical indicators revealing the optimal time lag (the number of time steps) between the initial conditions and the evolved pattern. Clearly, the criteria for the optimality, and the structure of statistical indicators must be predetermined before any further decisions could be taken.

### 2.4. The Wada Index for the Evaluation of the Image Complexity

One of the statistical indicators used for the evaluation of the complexity of the self-organizing patterns in this paper is the Wada index. The Wada index is originally proposed to detect the existence of Wada boundaries in phase plots of nonlinear dynamical systems [30]. The Wada index is based on the truncated and weighted Shannon entropy [30]. And though the Shannon entropy is commonly used to evaluate the randomness of a digital grayscale image [31,32], it is shown in [30] that the Wada index is also capable to evaluate the complexity of the basin boundary–what makes it applicable for numerous problems in nonlinear dynamics and image processing in general.

The lakes of Wada are three (or more) disjoint connected open sets of the plane with the counterintuitive property that they all have the same boundary [33,34,35,36]. The Wada basins of a nonlinear system possessing several coexisting attractors can be visualised as a colormap where the color of the point in the phase space of the initial conditions corresponds to the attractor to which the system evolves from this initial condition. The Wada index introduced in [30] helps not only to distinguish fractal and Wada basins of attraction but also measure the randomness of the distribution of different colors in a phase plot represented as a color or grayscale digital image [30].

Let us list the following notations required for the introduction of the Wada index:

- *s*—the size of the border of a s×s square observation window measured in the number of pixels; s≥2.
- *m*—the number of different colors in the observation window; m≥1.
- $\nu_{k}$, k=1,2,…,m—the number of the *k*-th color pixels in the observation window.
- $p_{k}=\frac{\nu_{k}}{s^{2}}$, k=1,2,…,m—the discrete probability of the *k*-th color in the observation window.
- The indicator function $1_{2}^{\left(s\right)}$ is equal to 1 if the number of colors in the observation window is greater or equal than 2: $1_{2}^{\left(s\right)}=\left\{\begin{array}{c} 1,\;m\geq 2,\\ 0,\;m=1. \end{array}\right.$
- The indicator function $1_{3}^{\left(s\right)}$ is equal to 1 if the number of colors in the observation window is greater or equal than 3: $1_{3}^{\left(s\right)}=\left\{\begin{array}{c} 1,\;m\geq 3,\\ 0,\;m\leq 2. \end{array}\right.$
- The Shannon entropy of different colors in the observation window:
  (9) $$e^{\left(s\right)}\left(p_{1},p_{2},\ldots ,p_{m}\right)=-\sum_{k=1}^{m}p_{k}\log\left(p_{k}\right).$$

The Wada index $\omega^{\left(s\right)}$ in the s×s observation window reads [30]:

(10) $$\begin{array}{c} \omega^{\left(s\right)}\left(p_{1},p_{2},\ldots ,p_{m}\right)=\frac{m}{\log\left(m\right)}1_{3}^{\left(s\right)}e^{\left(s\right)}=\left\{\begin{array}{c} 0,\;m<3,\\ -\frac{m}{\log\left(m\right)}\sum_{k=1}^{m}p_{k}\log\left(p_{k}\right),\;m\geq 3. \end{array}\right. \end{array}$$

The Wada index $W^{\left(s\right)}$ for the whole digital digital image reads [30]:

(11) $$W^{\left(s\right)}=\frac{\sum_{k=1}^{N}\omega_{k}^{\left(s\right)}}{\sum_{k=1}^{N}1_{2,k}^{\left(s\right)}},$$

where $\omega_{k}^{\left(s\right)}$ and $1_{2,k}^{\left(s\right)}$ is the Wada index $\omega^{\left(s\right)}$ and the indicator function $1_{2}^{\left(s\right)}$ in the *k*-th observation window. The size of the observation window *s* is set to 16 in all further computations because 16×16=256 results exactly into the total number of different levels of brightness in a grayscale image.

### 2.5. Other Statistical Indicators for the Evaluation of the Image Complexity

It is interesting to observe that the mean of the brightness of pixels in the self-organizing pattern does not necessarily coincide with 127.5 (the average between black (0) and white (255)). Thus, the second statistical indicator used in this study $\bar{N}$ is the mean of the brightness of pixels in the pattern of preys.

**Figure 2:**
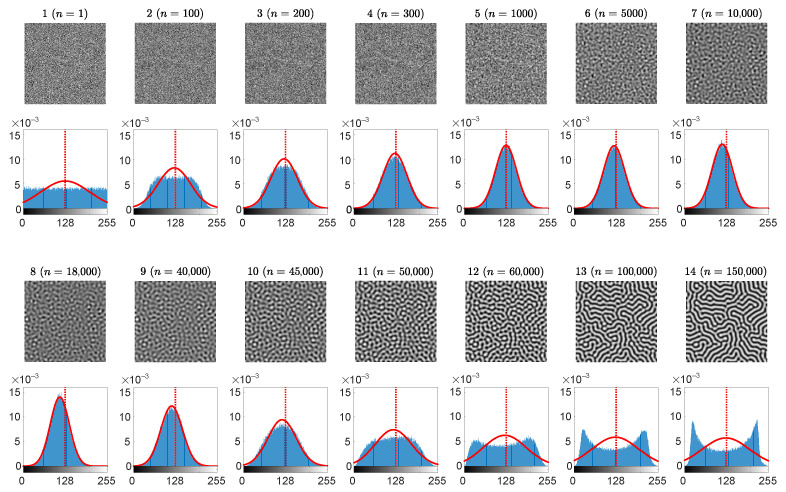
The evolution of the self-organizing pattern of preys. The snapshots of the partially developed patterns at 14 different time steps n= 1, 100, 200, 300, 1000, 5000, 10,000, 18,000, 40,000, 45,000, 50,000, 60,000, 100,000, 150,000 are enumerated from 1 to 14 (the 1st and the 3rd rows). The distribution histogram of each individual snapshot is shown in blue (the 2nd and the 4th rows). The best approximating Gaussian distribution curve is plotted in red line on top of the histogram. Vertical red dashed lines correspond to the center point between black and white.

Initial computational experiments with the evolution of self-organizing patterns (Figure 2) show that the distribution of the grayscale brightness of pixels does experience radical transformations from a uniform distribution to an arc-sine type distribution. The distribution becomes almost a normal distribution in the middle of the transient processes (Figure 2). Therefore, the third statistical indicator used in this paper is the *p*-value of the Kolmogorov-Smirnov criterion for the normality testing of the distribution function.

Finally, the similarity between the original dichotomous secret image and the reconstructed dichotomous secret image can be computed as a correlation coefficient ρ between these two binary images. Note that the values of Pearson, Spearman and Kendall’s tau correlation coefficients coincide if both variables are dichotomous. The phi coefficient, which is a measure of association for two binary variables, also yields the same value as Pearson, Spearman and Kendall’s tau correlation coefficients [37].

## 3. Results and Discussion

### 3.1. Optimal Information Hiding in Stripe-Type Patterns

The set of the parameters r=0.5, ε=1, β=0.6, K=2.6, η=0.25, w=0.4, B=0.3846, $D_{11}=0.01$, $D_{12}=0.0788$, $D_{21}=0.01$, $D_{22}=1$, $L_{x}=L_{y}=80$, h=0.25, τ=0.01 in the BDA predator-prey model results in a stripe-type pattern of preys [28]. Partially developed patterns of preys are shown at 14 different time steps (the 1st and the 3rd rows in Figure 2). The distribution histogram of the brightness for each individual snapshot is shown in blue (the 2nd and the 4th rows in Figure 2). The best approximating Gaussian distribution curve is plotted in overlapping red lines on top of the histograms. The vertical red dashed lines correspond to the center point between black and white. Note that the center point 127.5 does not necessarily coincide with the mean of the brightness of the pattern $\bar{N}$.

As mentioned previously, the small scale spatial chaos at the first snapshot is evolving to the large scale spatial chaos at the end of the computational experiment. At the beginning, the distribution of the self-organizing pattern is uniform on the interval [0,255] (Figure 2). It is interesting to observe that this distribution gradually becomes similar to the Gaussian distribution, and finally it evolves into an arcsine-type distribution. The similarity to the arcsine-type distribution can be explained by the fact that fully evolved pattern of stripes consists of two dominating colors–black and white. Therefore, the distribution histogram has two peaks located near the ends of the interval.

**Figure 3:**
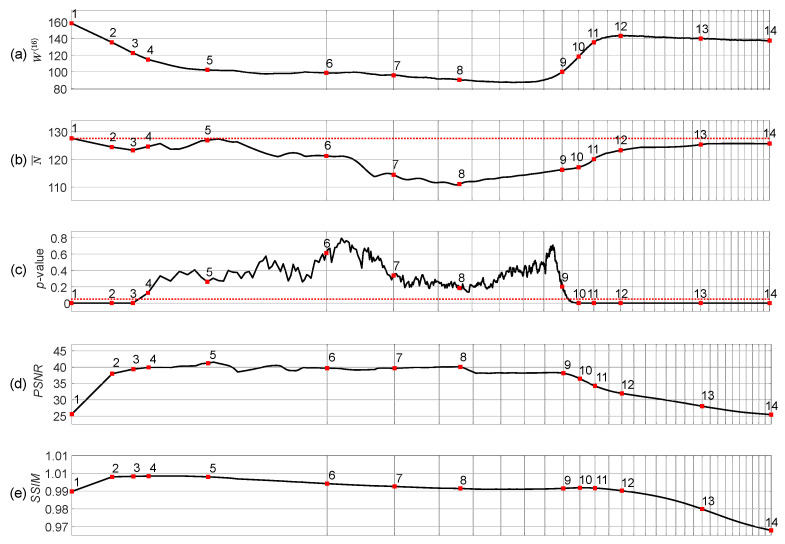
Statistical characteristics of the evolution of the self-organizing pattern of preys. The complexity of the self-organizing pattern is evaluated at each time step by computing the Wada index $W^{\left(16\right)}$ (panel (**a**)). The mean of the brightness of the pattern $\bar{N}$ is shown in panel (**b**). The red horizontal dotted line in panel (**b**) denotes the average between black and white. The *p*-value of the Kolmogorov-Smirnov criterion for the normality testing of the distribution function is depicted in panel (**c**). The red horizontal dotted line in panel (**c**) denotes the significance level equal to 0.05. Red squares enumerated from 1 to 14 correspond to the appropriate snapshots of the pattern during its evolution (Figure 2). PSNR and SSIM computed for the patterns with and without the embedded secret information are depicted respectively in panels (**d**,**e**). The scale of the *x*-axis is set to $\log^{3}n$; one interval marked by a gray vertical line corresponds to 5000 discrete time steps.

The complexity of the self-organizing pattern of preys is evaluated at each time step of the evolution process by computing the Wada index $W^{\left(16\right)}$. Also, the mean of the brightness of the pattern $\bar{N}$, and the *p*-value of the Kolmogorov-Smirnov criterion for the normality testing of the distribution function are computed during the whole evolution process (Figure 3).

Starting from the beginning, we see that the Wada index $W^{\left(16\right)}$ is relatively high (Figure 3a). Then $W^{\left(16\right)}$ drops down and rises again when the stripe-type pattern gets fully developed. After the initial drop, a quasi-stationary state can be observed between the 5th and the 9th snapshots.

Note that $\bar{N}$ drops below 127.5 in the middle of the self-organization process (Figure 3b). Figure 3c depicts the variation of the *p*-value of the Kolmogorov-Smirnov criteria which is used to test if the brightness of the self-organizing pattern is normally distributed. The null hypothesis states that the data comes from a standard normal distribution, against the alternative hypothesis that it does not come from such a distribution. The red dotted horizontal line in Figure 3c stands for the significance level of 0.05. Red squares (enumerated from 1 to 14) in Figure 3c denote the appropriate snapshots in the evolution of the self-organizing pattern (Figure 2). When the *p*-value is less than the significance level at the beginning and at the end of the evolution (snapshots 1–3 and 10–14) the null hypothesis is rejected and the data favors the alternative hypothesis. When the *p*-value is greater than the significance level (the middle part of the evolution including snapshots 4–9), we fail to reject the null hypothesis that data comes from the normal distribution.

The peak signal-to-noise ratio (PSNR) and structural similarity index (SSIM) are computed for the patterns with and without the embedded secret information during the whole evolution process of the patterns (Figure 3d–e). Values of the PSNR reaching approximately 40 dB (for the snapshots 4–8) and the values of the SSIM approaching 1 (snapshots 2–5) indicate that the perturbation of the initial conditions by the dot skeleton mask of the secret information do not significantly change the developed self-organizing pattern.

We seek to find out the time interval where the information hiding algorithms do perform in the optimal way. In other words, the target function describing the optimality of the image hiding algorithm must be defined.

**Figure 4:**
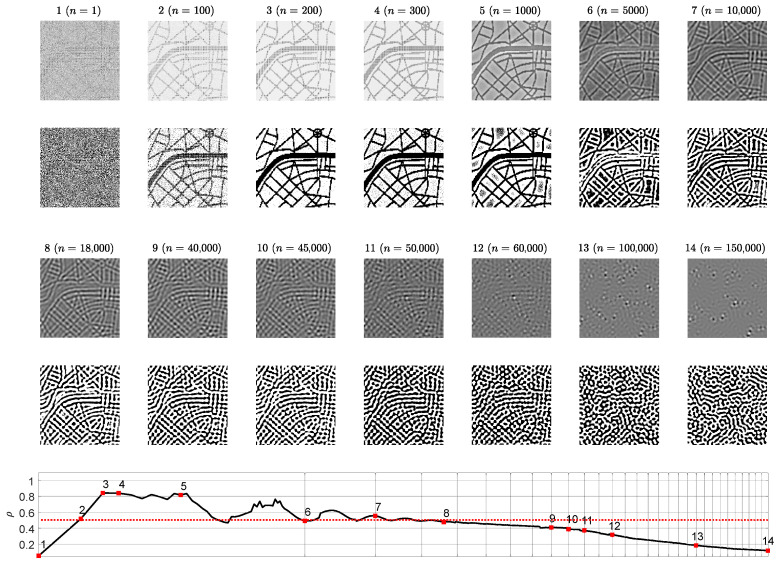
Difference images at different times steps *n* (1st and 3rd rows) and the corresponding dichotomous images (2nd and 4th rows). The correlation coefficient ρ reveals the statistical similarity between the original secret image and a dichotomous decoded secret image at time step *n*. Value ρ>0.5 corresponds to the clear interpretability of the decoded secret. The scale of the *x*-axis is set to $\log^{3}n$; one interval marked by a gray vertical line corresponds to 5000 discrete time steps.

The contrast enhanced difference images revealing the reconstructed secret information are depicted on the 1st and on the 3rd rows of Figure 4. The below counterparts depict the dichotomous representation of each difference image (the 2nd and 4th rows of Figure 4). The correlation coefficient ρ serves as a similarity indicator between the original secret image and the reconstructed dichotomous secret image. If ρ>0.5 then a human eye is still capable to interpret the secret image (the last row of Figure 4).

The presented results in Figure 2 and Figure 3 help to reach the following conclusion. The image hiding algorithm based on the difference between the self-organizing pattern started from random initial conditions and the pattern started from the same initial conditions perturbed by the dot-skeleton representation of the secret image does not work well if the stopping criterion for the evolution is not clearly defined.

If the set of the parameters defining the evolution of the pattern yield the pattern of stripes (Figure 2), then the stopping criterion must be selected according to the following three conditions:

- The Wada index $W^{\left(16\right)}$ should drop down from the initial value and should get stabilized before growing back again.
- The mean of the brightness of the pattern $\bar{N}$ should remain around the average between black and white before dropping down significantly below the average.
- The *p*-value of the Kolmogorov-Smirnov criterion should grow above 0.05 what indicates that the distribution becomes Gaussian.

### 3.2. Optimal Information Hiding in Patterns of Spots

Computational experiments are continued with the BDA predator-prey model, but with a different set of the parameters. For example, r=0.5, ε=1, β=0.6, K=2.6, η=0.25, w=0.4, B=0.4846, $D_{11}=0.01$, $D_{12}=-0.0269$, $D_{21}=0.01$, $D_{22}=1$, $L_{x}=L_{y}=80$, h=0.25, τ=0.01 yield the pattern of spots instead of the stripe-type pattern [28].

**Figure 5:**
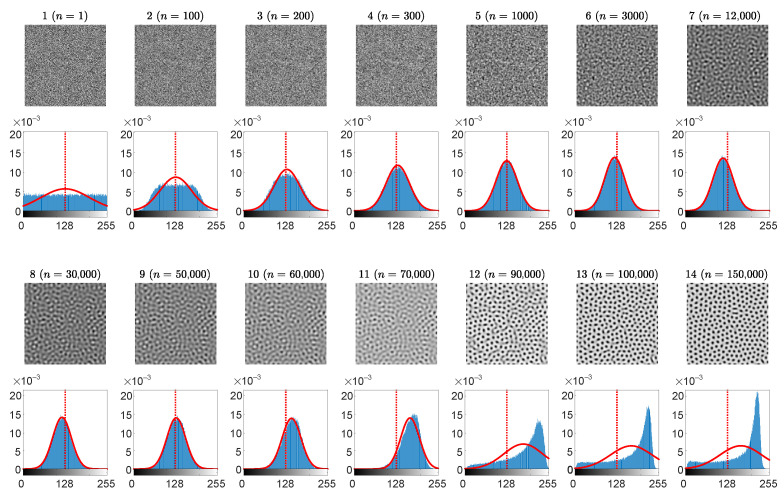
Information hiding in patterns of spots. The evolution of the self-organizing pattern of preys. The snapshots of the partially developed patterns at 14 different time steps n= 1, 100, 200, 300, 1000, 3000, 12,000, 30,000, 50,000, 60,000, 70,000, 90,000, 100,000, 150,000 are enumerated from 1 to 14 (the 1st and the 3rd rows). The distribution histogram of each individual snapshot is shown in blue (the 2nd and the 4th rows). The best approximating Gaussian distribution curve is plotted in red line on top of the histogram. Vertical red dashed lines correspond to the center point between black and white.

Partially developed self-organizing patterns of preys are presented at 14 different time steps (the 1st and the 3rd rows in Figure 5). The distribution histogram of the brightness for each individual snapshot is shown in blue (the 2nd and the 4th rows in Figure 5). The best approximating Gaussian distribution curve is plotted in red. The vertical red dashed lines correspond to the center point between black and white (which does not necessarily coincide with the mean of the brightness of the pattern $\bar{N}$).

At the beginning, the distribution of the pattern of preys is uniform on the interval [0,255], then it gradually becomes similar to the Gaussian distribution, and finally this distribution evolves into a left-skewed distribution (Figure 5). The negative skewness can be explained by the fact that the bright background dominates over the dark spots in the fully evolved pattern of preys.

**Figure 6:**
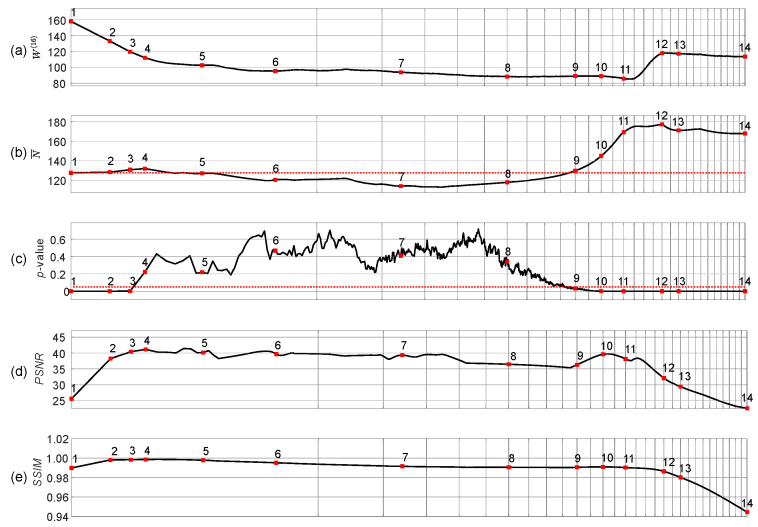
Information hiding in patterns of spots. Statistical characteristics of the evolution of the self-organizing pattern of preys. The complexity of the self-organizing pattern is evaluated at each time step by computing the Wada index $W^{\left(16\right)}$ (panel (**a**)). The mean of the brightness of the pattern $\bar{N}$ is shown in panel (**b**). The red horizontal dotted line in panel (**b**) denotes the average between black and white. The *p*-value of the Kolmogorov-Smirnov criterion for the normality testing of the distribution function is depicted in panel (**c**). The red horizontal dotted line in panel (**c**) denotes the significance level equal to 0.05. Red squares enumerated from 1 to 14 correspond to the appropriate snapshots of the pattern during its evolution (Figure 5). PSNR and SSIM computed for the patterns with and without the embedded secret information are depicted respectively in panels (**d**,**e**). The scale of the *x*-axis is set to $\log^{3}n$; one interval marked by a gray vertical line corresponds to 5000 discrete time steps.

The Wada index $W^{\left(16\right)}$, the mean of the brightness of the pattern $\bar{N}$, the *p*-value of the Kolmogorov-Smirnov criterion for the normality testing of the distribution function, the peak signal-to-noise ratio and the structural similarity index during the whole evolution process are shown in Figure 6.

**Figure 7:**
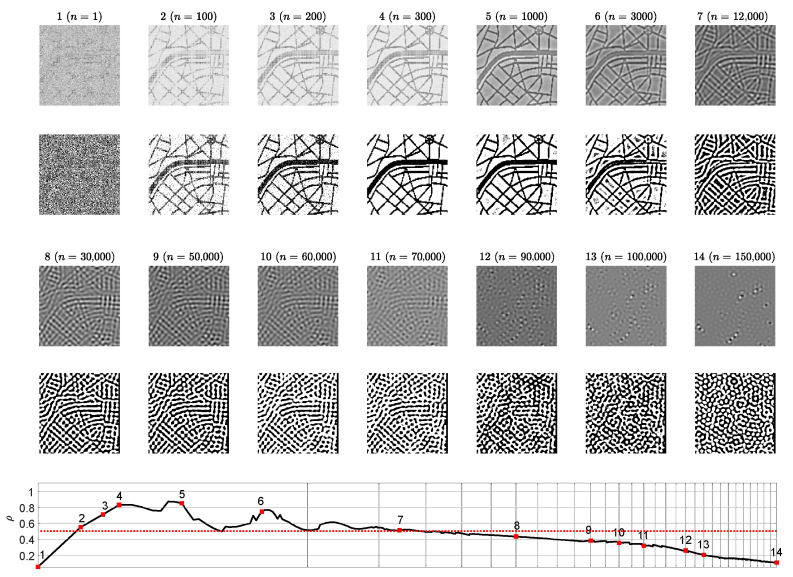
Information hiding in patterns of spots. Difference images at different times steps *n* (1st and 3rd rows) and the corresponding dichotomous images (2nd and 4th rows). The correlation coefficient ρ reveals the statistical similarity between the original secret image and a dichotomous decoded secret image at time step *n*. Value ρ>0.5 corresponds to the clear interpretability of the decoded secret. The scale of the *x*-axis is set to $\log^{3}n$; one interval marked by a gray vertical line corresponds to 5000 discrete time steps.

The reconstructed secret images and their dichotomous representations are depicted on the 1st–4th rows of Figure 7. The correlation coefficient ρ (the last graph in Figure 7) reveals the level of the similarity between the original secret image and the reconstructed dichotomous secret image. A human eye is still capable to interpret the secret image when ρ>0.5 (Figure 7).

It is possible to conclude that the stopping criterion for the information hiding algorithm based on the generation of patterns of spots does meet the same conditions as listed in Section 3.1 for the stripe-type patterns.

### 3.3. Optimal Information Hiding in Unstable Patterns

**Figure 8:**
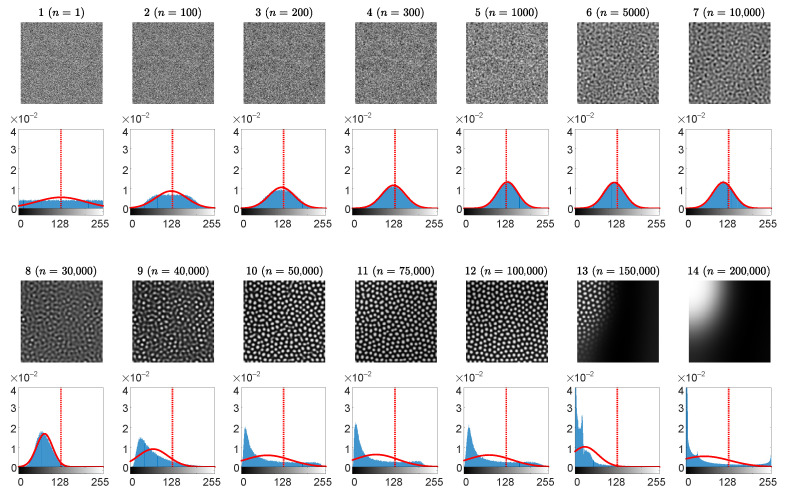
Information hiding in unstable patterns. The evolution of the self-organizing pattern of preys. The snapshots of the partially developed patterns at 14 different time steps n= 1, 100, 200, 300, 1000, 5000, 10,000, 30,000, 40,000, 50,000, 75,000, 100,000, 150,000, 200,000 are enumerated from 1 to 14 (the 1st and the 3rd rows). The distribution histogram of each individual snapshot is shown in blue (the 2nd and the 4th rows). The best approximating Gaussian distribution curve is plotted in red line on top of the histogram. Vertical red dashed lines correspond to the center point between black and white.

When cross-diffusion coefficients satisfy the conditions $D_{21}=0$ and $D_{12}\neq 0$ or, biologically speaking, when preys move towards the higher concentration of predators, and the predators move along their own concentration gradient, the equilibrium state becomes unstable [28]. Such a situation can be illustrated by computational experiments with a following set of parameters: r=0.5, ε=1, β=0.6, K=2.6, η=0.25, w=0.4, B=0.2769, $D_{11}=0.01$, $D_{12}=0.1920$, $D_{21}=0$, $D_{22}=1$, $L_{x}=L_{y}=80$, h=0.25, τ=0.01 [28]. Partially developed self-organizing patterns of preys are presented at 14 different time steps (the 1st and the 3rd rows in Figure 8). Snapshots 7–9 reveal the competition between spots and stripes, then the pattern of spots begins to settle down (snapshots 10–12) and, finally, the wave pattern emerges (snapshots 13–14). The distribution histogram of the brightness of each individual snapshot is shown in blue (the 2nd and the 4th rows in Figure 8). The best approximating Gaussian distribution curve is plotted in red.

At the beginning of the evolution, the distribution of the pattern of preys is uniform on the interval [0,255] (Figure 8). Then it gradually becomes similar to the Gaussian distribution, and finally evolves into an right-skewed distribution. The positive skewness is a result of the dark background, which dominates over white spots.

**Figure 9:**
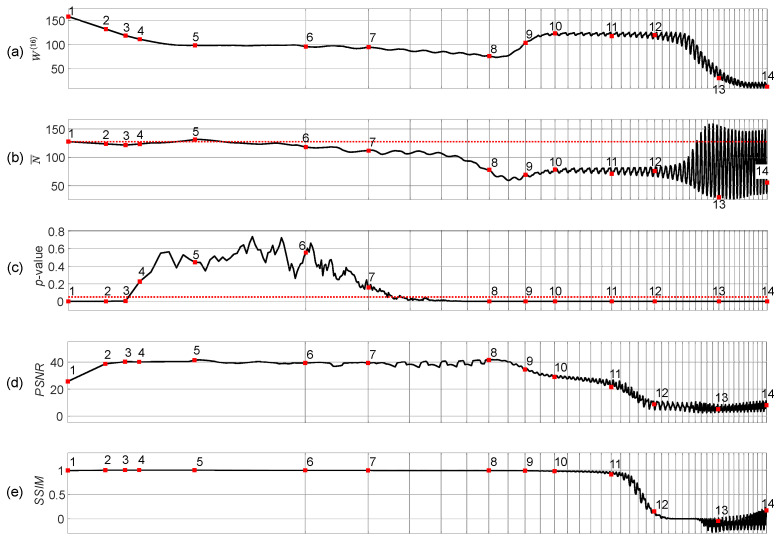
Information hiding in unstable patterns. Statistical characteristics of the evolution of the self-organizing pattern of preys. The complexity of the self-organizing pattern is evaluated at each time step by computing the Wada index $W^{\left(16\right)}$ (panel (**a**)). The mean of the brightness of the pattern $\bar{N}$ is shown in panel (**b**). The red horizontal dotted line in panel (**b**) denotes the average between black and white. The *p*-value of the Kolmogorov-Smirnov criterion for the normality testing of the distribution function is depicted in panel (**c**). The red horizontal dotted line in panel (**c**) denotes the significance level equal to 0.05. Red squares enumerated from 1 to 14 correspond to the appropriate snapshots of the pattern during its evolution (Figure 8). PSNR and SSIM computed for the patterns with and without the embedded secret information are depicted respectively in panels (**d**,**e**). The scale of the *x*-axis is set to $\log^{3}n$; one interval marked by a gray vertical line corresponds to 5000 discrete time steps.

The Wada index $W^{\left(16\right)}$, the mean of the brightness of the pattern $\bar{N}$, and the *p*-value of the Kolmogorov-Smirnov criterion for the normality testing of the distribution function during the whole evolution process are shown in Figure 9. At the beginnig of the evolution, the Wada index $W^{\left(16\right)}$ is relatively high, then it drops down and reaches quasi-stationary state, then rises again, but begins to oscillate and finally drops almost down to zero. The mean of the brightness of the pattern $\bar{N}$ at the end of the demonstrated evolution also exhibits oscillations with an increasing amplitude. The values of PSNR and SSIM computed for the patterns with and without the embedded secret information indicate that these patterns do not differ significantly until n=30,000 (Figure 9d–e).

**Figure 10:**
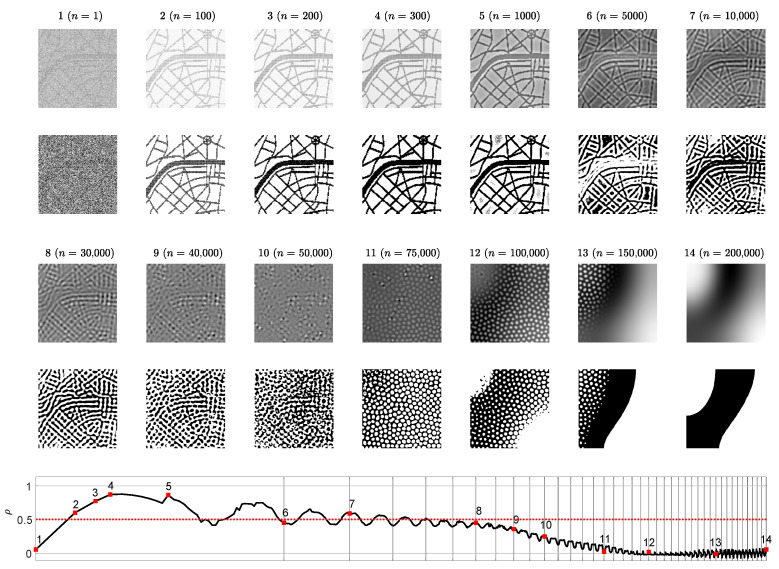
Information hiding in unstable patterns. Difference images at different times steps *n* (1st and 3rd rows) and the corresponding dichotomous images (2nd and 4th rows). The correlation coefficient ρ reveals the statistical similarity between the original secret image and a dichotomous decoded secret image at time step *n*. Value ρ>0.5 corresponds to the clear interpretability of the decoded secret. The scale of the *x*-axis is set to $\log^{3}n$; one interval marked by a gray vertical line corresponds to 5000 discrete time steps.

The reconstructed secret images and their dichotomous representations are depicted on the 1st–4th rows of Figure 10, the correlation coefficient ρ is given in the last graph in Figure 10. Variation of $W^{\left(16\right)}$, $\bar{N}$, *p*-value and ρ during the evolution of the unstable pattern of white spots leads to the same conditions for the stopping criterion as in Section 3.1 and Section 3.2.

### 3.4. The Robustness of the Proposed Scheme

**Figure 11:**
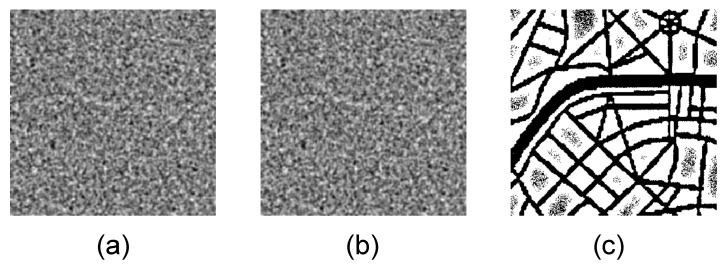
Self-organizing patterns with and without the embedded secret information at n=1000 iterations are depicted in panels (**a**,**b**) respectively. Dichotomous representation of the difference between patterns in panels (**a**,**b**) reveals the secret image in panel (**c**).

The self-organizing pattern generated by the BDA model using the set of the parameters listed in Section 3.1 after n=1000 iterations (Figure 2, n=1000) is used to demonstrate the robustness of the proposed image hiding scheme to the rotation and cropping, partial destruction, contamination by noise, and steganalysis algorithms. Self-organizing patterns with and without the embedded secret information at n=1000 iterations are presented in Figure 11a,b. The dichotomous representation of the difference between patterns in panels (a) and (b) reveals the secret image (panel (c)).

**Figure 12:**
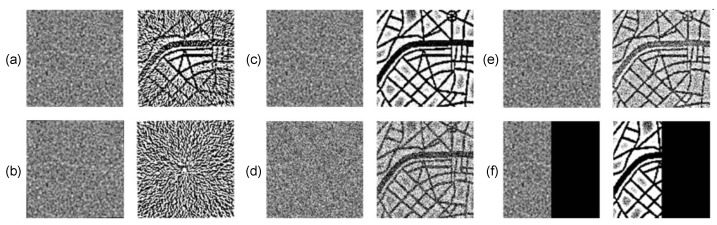
The robustness of the proposed scheme to the rotation and cropping, the contamination by noise and partial destruction of the image is demonstrated for the pattern with the embedded secret information at n=1000 iterations (Figure 11a). The rotation of the pattern by 0.1° and 1° and cropping (panels (**a**,**b**) on the left) destroy the secret information (panels (**a**,**b**) on the right). Contamination of the pattern by the salt and pepper noise partially destroys the decoded image in panels (**c**,**d**) (5% of pixels of the pattern are affected in panel (**c**) and 50% of pixels–in panel (**d**)) Contamination of the pattern by Gaussian noise with zero mean and standard deviation equal to 0.0001 affects approximately 85% of pixels of the pattern (panel (**e**) on the left). A partial destruction of the pattern (panel (**f**) on the left) enables to retrieve the secret information only within the undamaged part (panel (**f**) on the right).

Patterns rotated by 0.1° and 1° and cropped are shown in Figure 12a,b (the images on the left). It is clear that the information decoded from the rotated images (even rotated by small angles) is fully destroyed (Figure 12a,b). Figure 12c,d reveals that the addition of the salt and pepper noise to the pattern with the embedded secret information partially destroys the decoded image. Note that 5% of pixels of the pattern with the embedded secret information are affected by the salt and pepper noise in panel (c) and 50% of pixels—in panel (d). However, the decoded secret image is still interpretable (Figure 12c,d). The contamination of the pattern by the Gaussian noise with zero mean and standard deviation equal to 0.0001 affects approximately 85% of pixels of the pattern but does not fully destroy the decoded image (Figure 12e). Note that a partial destruction of the pattern (Figure 12f) enables to retrieve the secret information only within the undamaged part of the image.

It is important to ensure that the secret information embedded into the self-organising pattern would not be discoverable by the eavesdropper by means of a straightforward analysis of the transmitted pattern. We demonstrate that the bit plane analysis and the RS analysis algorithms (standard steganalysis techniques) are unable to reveal the fact that any secret information is concealed in the self-organizing pattern.

**Figure 13:**
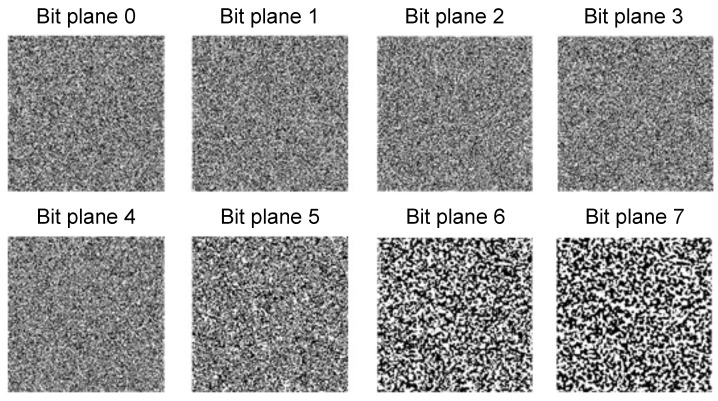
Bit planes of the pattern with the embedded secret information in Figure 11a do not reveal the secret information.

Figure 13 demonstrates that the bit planes of the pattern with the embedded image (depicted in Figure 11a) reveal no secret information.

**Figure 14:**
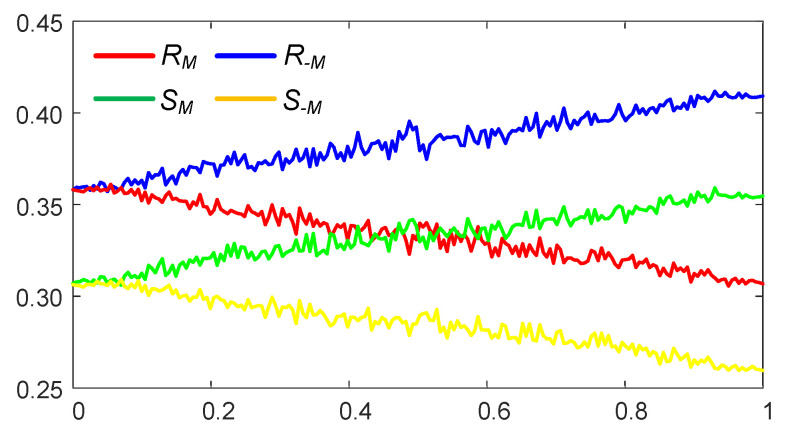
The RS diagram computed for the pattern with the embedded secret information in Figure 11a shows that the proposed image hiding algorithm is robust to the RS steganalysis. The *x*-axis is the percentage of pixels with flipped LSBs; the *y*-axis is the relative number of regular and singular groups with masks *M* and −M). Note that $R_{M}≅R_{-M}$ and $R_{S}≅R_{-S}$ at x=0.

The RS analysis algorithm tests the robustness of the proposed algorithm to the statistical steganalysis [38]. The number of regular groups for mask *M* is $R_{M}$ (the percentage of all groups). Similarly, $S_{M}$ is the relative number of singular groups. $R_{-M}$ and $S_{-M}$ reveal the numbers of regular and singular groups respectively for the negative mask −M. The statistical hypothesis of the RS analysis implies that a typical image without the embedded information (corresponding to the zero point on the *x*-axis) does produce approximately equal expected values of $R_{M}$ and $R_{-M}$ (the same holds for $S_{M}$ and $S_{-M}$) [38]. The RS diagram computed for the pattern with the embedded secret information is presented in Figure 14. The *x*-axis is the percentage of pixels with flipped LSBs; the *y*-axis is the relative number of regular and singular groups with masks *M* and −M respectively. Note that $R_{M}≅R_{-M}$ and $R_{S}≅R_{-S}$ at x=0. The RS diagram indicates that the proposed image hiding algorithm based on the self-organising patterns is robust to the RS steganalysis.

### 3.5. More Examples of Different Carrier Patterns and Hidden Images

**Figure 15:**
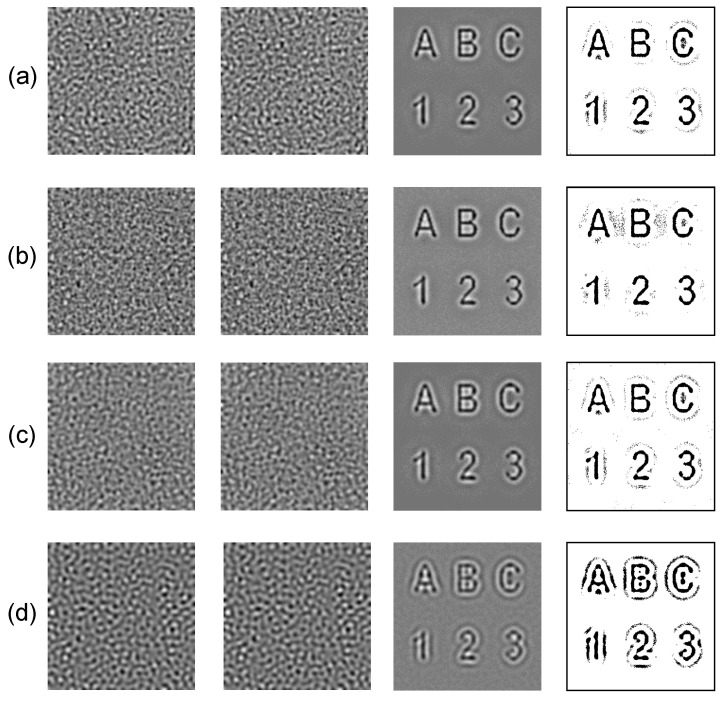
Hiding alphanumerical symbols in the patterns generated by the BDA model: the set of the parameters defined in Section 3.1 with n=4900 is used in panel (**a**); the set of the parameters defined in Section 3.2 with n=2700 is used in panel (**b**); the set of the parameters defined in Section 3.3 with n=5700 is used in panel (**c**); the set of the parameters defined in Section 3.2 with n=13,900 is used in panel (**d**). Self-organizing patterns with and without the embedded secret information are depicted in the 1st and the 2nd columns; the decoded secret images and their dichotomous counterparts are shown in the 3rd and the 4th columns.

**Figure 16:**
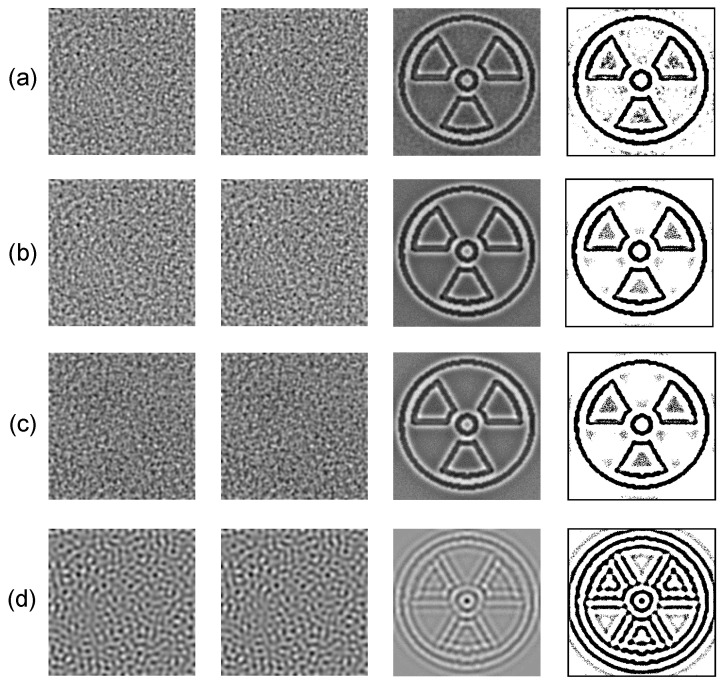
Hiding the radiation sign in the patterns generated by the BDA model: the set of the parameters defined in Section 3.1 with n=2600 is used in panel (**a**); the set of the parameters defined in Section 3.2 with n=2500 is used in panel (**b**); the set of the parameters defined in Section 3.3 with n=2700 is used in panel (**c**); the set of the parameters defined in Section 3.2 with n=18,400 is used in panel (**d**). Self-organizing patterns with and without the embedded secret information are depicted in the 1st and the 2nd columns; the decoded secret images and their dichotomous counterparts are shown in the 3rd and the 4th columns.

The proposed steganography algorithm based on the BDA model with the self- and cross-diffusion is demonstrated for different secret images. Three different sets of parameters of the BDA model are used to hide each secret image. Modelling results are given in Figure 15 and Figure 16. All self-organizing patterns with the embedded secret information in Figure 15 and Figure 16 look rather similar, in spite of the fact that these patterns are generated by the BDA model with the different sets of the parameters. Note that the further evolution of these patterns would result into the distinct patterns of stripes, dots or unstable patterns (the BDA model with the self- and cross-diffusion cannot produce other types of patterns [28]). This similarity is predetermined by the derived stopping criterion (Section 3.1) which does not allow the patterns to become fully-developed. The reconstructed secret image becomes distorted if the stopping condition exceeds the optimal stopping criterion (Figure 15d and Figure 16d).

## 4. Concluding Remarks

Information hiding in self-organizing patterns is discussed in this paper. The hiding scheme is implemented in the form of a difference image between two patterns. The first pattern is allowed to evolve from random initial conditions. The second pattern is started to evolve from the same random initial conditions but perturbed by the dot-skeleton representation of the secret image. Nonlinear self- and cross-diffusion effects in the BDA model are exploited to generate smooth representations of the secret image in the difference image.

However, the quality of the reconstructed secret image does depend on the stopping criterion which defines the duration of the evolution of two patterns. The main objective of this paper is to experimentally define the statistical features of self-organizing patterns which can yield optimal representations of the secret image. Moreover, it is well known in the scientific literature that the BDA model is capable to generate different patterns. Patterns of stripes, patterns of spots, or even unstable patterns are typical patterns generated by the BDA model at different values of its parameters.

It appears that the optimal duration of the pattern evolution does not depend on the type of the pattern. Three main conditions must be satisfied before the evolution of the pattern could be stopped. The Wada index of the evolving pattern must drop from the initial higher values–the small scale chaos represented by the random initial conditions should be transformed into a smoother image. The mean brightness of the image should remain around the middle of the brightness interval before dropping down significantly below the average. Finally, the distribution function should remain Gaussian.

All three conditions must hold true simultaneously. Those conditions do specify the state of the developing pattern when the small scale chaos of the initial random distribution is gradually transforming into the large scale chaos of the primitives (stripes or spots) in the fully developed pattern. As mentioned previously, it is rather unexpected to observe that the conditions do not depend on the type of the pattern in the BDA model.

Although the concept of image hiding in self-organizing patterns has been introduced more than a decade ago, the discussion on the time required for the pattern to evolve before the hidden image can be delivered to the recipient has been missing in the scientific literature. It appears, that many important factors do contribute to the formulation of the stopping conditions (the type of the distribution function, the Wada index, the mean of the brightness). Moreover, it appears that the formulated stopping criteria do not depend on the type of the pattern generated by the BDA predator-prey model (even if the self-organizing pattern becomes unstable). These important aspects do constitute the novelty of this work.

Clearly, the BDA model is only one of many models used to generate self-organizing patterns. Experimental (computational) observation of the conditions yielding optimal information hiding in self-organizing patterns generated by other types of models remains a definite objective of future research.

The discussed image hiding algorithm is based on nonlinear physical interactions during the formation of self-organizing patterns. The secret image is interpreted as a dichotomous image comprised from relatively thick lines or spots. It is natural to compare the functionality of this image hiding algorithm with other similar image hiding algorithms. One of the closest algorithms (in terms of the structure of the secret image and the information hiding capacity) is the image hiding scheme based on dynamic visual cryptography (DVC) [39,40]. The DVC scheme is also based on nonlinear physical processes (the formation of time-averaged moiré fringes in the oscillating carrier image). The secret image is also a dichotomous image. The information hiding capacity of the DVC scheme is comparable to the one discussed in this paper (it is determined by the wavelength of the moiré grating).

A clear advantage of the DVC scheme is that the dichotomous secret is revealed in the time-averaged carrier image (the computation of the difference image between two patterns is not required) [39,40]. A serious disadvantage of the DVC scheme is the security of the cover image (a naked human eye cannot see the secret image—but statistical algorithms can reveal the embedded secret) [39,40]. This comparison is a good example of the well-known “No free lunch” theorem [41,42]. It is very difficult to expect that a single scheme would have all possible advantages, compared with other existing schemes. Anyway, the development of an image hiding algorithm with the best features inherited from the BDA model and the DVC scheme remains a definite objective of future research.

## Data Availability

The data presented in this study are available on request from the corresponding author.
